# Nitrogen Partial Pressure-Controlled Deposition of TiMoSiN Coatings via Arc Ion Plating: Mechanical, Tribological, and Corrosion-Resistant Properties

**DOI:** 10.3390/ma19112196

**Published:** 2026-05-23

**Authors:** Jibo Huang, Ting Yang, Cheng Zhou, Zhaoguo Qiu

**Affiliations:** 1 School of Automation, Guangdong Polytechnic Normal University, Guangzhou 510665, China; jibohuang@foxmail.com; 2 China-Mongolia Belt and Road Joint Laboratory of Mineral Processing Technology, Inner Mongolia Academy of Science and Technology, Hohhot 010000, China; 3 School of Materials Science and Engineering, South China University of Technology, Guangzhou 510640, China; 202021021909@mail.scut.edu.cn

**Keywords:** TiMoSiN coating, arc ion plating, nitrogen partial pressure, mechanical properties, tribological properties, corrosion resistance

## Abstract

TiN coatings have been widely employed in cutting tools due to their high hardness and excellent wear resistance. While most research on nitride coatings has focused on binary (e.g., TiN) and ternary (e.g., TiAlN, TiSiN) systems, the quaternary TiMoSiN system remains comparatively underexplored. In response to the growing demand for comprehensive coating performance under increasingly complex working conditions, this work incorporates Mo and Si into the TiN system to synergistically enhance mechanical, tribological, and corrosion-resistant properties. TiMoSiN coatings were deposited onto cemented carbide substrates by arc ion plating using a Ti_0.8_Mo_0.1_Si_0.1_ alloy target. The influence of nitrogen partial pressure (0.2–1.7 Pa) on the microstructure, mechanical properties, tribological behavior, and electrochemical corrosion performance was investigated. The results show that nitrogen partial pressure plays a critical role in regulating the chemical composition, phase structure, and preferred orientation of the coatings. As the nitrogen partial pressure increases, surface macroparticles are reduced, while the Ti and Mo contents decrease and the Si and N contents increase. The phase structure evolves from a dual-phase mixture of TiN and Ti_2_N to a single TiN phase, accompanied by a shift in preferred orientation from (111) to (200). The hardness of the coatings ranges from 36.2 to 43.1 GPa, reaching a maximum of 43.1 GPa at 1.0 Pa. The coating deposited at 0.6 Pa exhibits the best overall performance: it achieves the lowest friction coefficient (0.349) and wear rate (1.08 × 10^−7^ mm^3^/(N·m)), together with the highest corrosion resistance, as reflected by the most noble corrosion potential (−152 mV) and the lowest corrosion current density (8.99 × 10^−8^ A·cm^−2^). This study demonstrates that nitrogen partial pressure effectively controls the microstructure and multifunctional properties of TiMoSiN coatings, providing practical process guidelines for their application in demanding cutting environments.

## 1. Introduction

Machining is a cornerstone of modern manufacturing, in which cutting tool performance is of paramount importance. Tool wear represents the predominant failure mode during material processing. The application of physical vapor deposition (PVD) techniques to deposit TiN coatings on cutting tools marked a significant advancement, substantially enhancing surface hardness and reducing wear [1,2,3].

In recent years, the drive toward lightweight design has increased industrial reliance on high-strength, low-density materials. For instance, aluminum alloys are now widely used in automotive manufacturing, titanium alloys in aerospace, and stainless steels in consumer goods. However, these materials are often classified as “difficult-to-machine” due to severe wear and work hardening during processing. Consequently, conventional TiN coatings struggle to meet the stringent demands of machining such materials, creating an urgent need for novel coatings with superior hardness and lower wear rates [4,5].

To enhance coating performance, multi-element alloying of TiN has been extensively explored. TiMoN coatings have attracted research interest due to their potential for reducing friction coefficients [6,7,8]. Studies indicate that Mo doping can induce solid-solution strengthening, increasing hardness, and may lead to the formation of self-lubricating MoO_3_ during friction, thereby lowering the friction coefficient [8,9]. Despite these advantages, the industrial application of TiMoN coatings remains limited. Challenges include insufficient hardness, relatively poor oxidation resistance (Mo_2_N oxidizes at around 350 °C), and an incomplete understanding of their wear mechanisms against different counterfaces [10].

While binary coatings such as TiN and CrN provide a foundation, their hardness, wear resistance, and oxidation resistance are often inadequate for advanced applications. Researchers have therefore pursued ternary and quaternary systems [11,12]. For example, doping TiN with Si or B significantly improves hardness and thermal stability, resulting in coatings such as TiSiN and TiBN [13]. Similarly, adding C or Mo enhances tribological performance, yielding low-friction TiCN or self-lubricating TiMoN coatings [8,14,15]. For tool and mold applications, multi-component nitride coatings, particularly those combining a hard matrix (e.g., TiN, CrN) with self-lubricating phases, are considered optimal. Such coatings offer an excellent balance of high hardness and superior tribological properties, making them ideal for machining difficult-to-cut materials [16,17].

Following early work on TiMoN coatings, which established relationships between composition, structure, hardness, and friction behavior, attention has turned to further improvements [8,18]. Doping with silicon (Si) is a promising strategy, as it is known to significantly enhance the hardness, thermal stability, and oxidation resistance of TiN-based coatings [19,20,21]. Therefore, the development of TiMoSiN quaternary coatings aims to synergistically combine the solid-solution and potential self-lubricating effects of Mo with the strengthening and anti-oxidation properties of Si [22,23]. Preliminary studies on TiMoSiN coatings, primarily fabricated by magnetron sputtering, confirm their potential for high hardness and low friction coefficients [22,23]. However, research remains limited, often focusing only on the effects of Mo or Si content on hardness and tribology at room temperature, with insufficient investigation into other critical properties such as adhesion and corrosion resistance. Moreover, the potential of arc ion plating (AIP), a technique known for high deposition rates and excellent coating adhesion, for fabricating TiMoSiN coatings has been underexplored, particularly regarding the systematic influence of key process parameters such as nitrogen partial pressure. In contrast to the extensive literature on binary and ternary nitride systems, the quaternary TiMoSiN composition remains comparatively rare in published studies.

To address these research gaps, this study employs arc ion plating to deposit TiMoSiN coatings onto cemented carbide (YG6x) substrates using a Ti_0.8_Mo_0.1_Si_0.1_ alloy target. The primary objective is to systematically investigate the effect of nitrogen partial pressure (varied from 0.2 to 1.7 Pa) on the coatings’ chemical composition, phase structure, preferred orientation, mechanical properties (hardness), tribological behavior (friction and wear rate), and electrochemical corrosion resistance. The goal is to identify the adequate synthesis parameters that coordinates microstructure and properties, thereby providing practical process guidelines for their application in demanding cutting environments, such as dry or minimum-quantity-lubrication machining of titanium alloys (e.g., Ti6Al4V), stainless steels, and high-strength aerospace components, as well as protective coatings for wear- and corrosion-prone molds and dies.

## 2. Materials and Methods

### 2.1. Coating Deposition

TiMoSiN coatings were deposited onto cemented carbide (YG6x, dimensions 15 mm × 15 mm× 4 mm) using an AIP-01 arc ion plating system (Shenyang Yuankehang Company, Shenyang, China). Prior to deposition, all substrates were ground and polished to a mirror finish, ultrasonically cleaned in anhydrous ethanol for 10 min, and subsequently dried and mounted on a planetary rotation fixture.

The deposition process employed a Ti_0.8_Mo_0.1_Si_0.1_ alloy target (purity 99.9%) for the TiMoSiN layer and a Cr target (purity 99.9%) for the interlayer. High-purity gases (Ar, N_2_, 99.99%) were used throughout the process.

The chamber was initially evacuated to a base pressure below 7.0 × 10^−3^ Pa, followed by substrate heating to 250 °C. Prior to deposition, the substrate surfaces were subjected to Ar^+^ ion bombardment cleaning using a Cr target (target current 75 A) under a bias voltage of −500 V for 5 min in an Ar atmosphere (0.2 Pa) to remove surface oxides and contaminants. Subsequently, a Cr adhesion layer was deposited for 10 min with a bias voltage of −100 V and target current of 75 A under an Ar pressure of 0.4 Pa. Cr was chosen as the interlayer material due to its excellent adhesion to both cemented carbide substrates and TiN-based coatings, as well as its widespread use in industrial arc ion plating processes.

After deposition of the Cr interlayer, the Cr target current was switched off. Subsequently, N_2_ was introduced while Ar was shut off, and TiMoSiN coatings were deposited using the Ti_0.8_Mo_0.1_Si_0.1_ alloy target (current 100 A) at a bias voltage of –150 V for 60 min. Nitrogen partial pressure was systematically varied (0.2, 0.6, 1.0, 1.4, and 1.7 Pa) to investigate its influence on coating properties. nitrogen partial pressure is a key process parameter in arc ion plating because it influences target nitriding, the degree of scattering of metal ions, and the energy/flux ratio of bombarding species. Low pressures typically favor strong ion bombardment and columnar growth but also more macroparticles, while high pressures reduce macroparticle density but may lower metal content and alter preferred orientation. The selected pressure range (0.2–1.7 Pa) covers these competing regimes, allowing systematic exploration of their effects. Detailed deposition parameters are summarized in Table 1.

### 2.2. Coating Characterization

Surface morphology, cross-sectional morphology and chemical composition of the as-deposited coatings were analyzed using a field-emission scanning electron microscope (FE-SEM, Nova NanoSEM 430, FEI, Eindhoven, The Netherlands) equipped with an energy-dispersive X-ray spectrometer (EDS, Oxford Instruments X-Max detector, Oxford Instruments, Abingdon, UK). EDS analysis was performed at an accelerating voltage of 15 kV, and quantification was carried out using standard ZAF correction.

The phase structure of the coatings was characterized by grazing incidence X-ray diffraction (GIXRD) using a SmartLab diffractometer (Rigaku Corporation, Akishima, Tokyo, Japan) with Cu Kα radiation (λ = 1.542 Å). The X-ray tube was operated at a voltage of 40 kV and a current of 40 mA. Diffraction patterns were recorded over a 2θ range of 10–90° with a step size of 0.01°. X-ray photoelectron spectroscopy (XPS) was performed using an Axis Supra spectrometer (Shimadzu/Kratos, Manchester, UK) to analyze the chemical bonding states of the coating deposited at 0.6 Pa. Raman spectroscopy was performed on the wear tracks of selected samples using a LabRAM Aramis confocal Raman spectrometer (Horiba France SAS, Palaiseau, France) equipped with a 532 nm (or 633 nm) laser as the excitation source. Spectra were recorded in the wavenumber range of 100–1000 cm^−1^ to identify the presence of oxide phases.

Hardness (H) and elastic modulus (E) of the coatings were evaluated by nanoindentation using a TTX-NHT3 nanoindenter (Anton Paar, Graz, Austria) equipped with a Berkovich diamond tip. The maximum load was set to 5 mN, with loading and unloading rates of 10 mN/min and a dwell time of 5 s at peak load. The Poisson’s ratio was assumed to be 0.3. To minimize measurement uncertainty, 8–10 indentations were performed on each sample, ensuring that the indentation depth remained below 10% of the coating thickness to avoid substrate interference. Average values are reported. Nanoindentation measurements were performed in accordance with ISO 14577-1:2015 [24].

Coating thickness was measured using a ball-cratering method (calotest) in accordance with JB/T 7707-1995 (2017) [25]. A steel ball (GCR15, diameter 30 mm) with diamond paste was rotated against the coated surface to form a spherical crater. The crater diameters were observed by optical microscopy, and the thickness was calculated using the formula:

t=(X×Y)/(2R)

where X and Y are the difference and sum of the outer and inner crater radii, and R is the ball radius. The deposition rate was obtained by dividing the thickness by the total deposition time (60 min).

### 2.3. Tribological Testing

**Figure 1:**
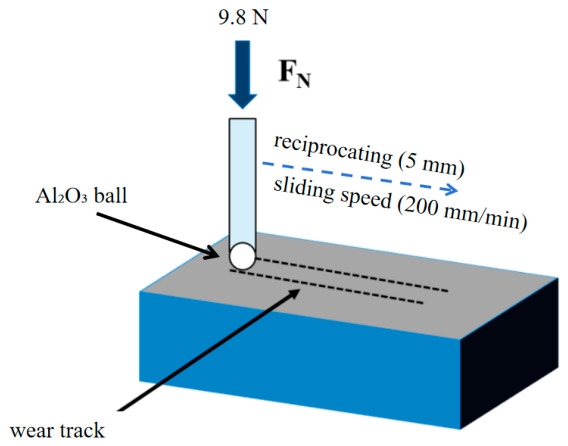
Schematic diagram of the reciprocating tribological test (MFT-4000).

Tribological behavior was evaluated using a reciprocating friction tester (MFT-4000, Lanzhou Huahui Instrument Technology Co., Ltd., Lanzhou, China) under ambient conditions (25 °C, relative humidity 40–50%), with reference to ASTM G99-23 (ball-on-flat configuration) [26]. A schematic diagram of the test setup is shown in Figure 1. An Al_2_O_3_ ball (diameter 4 mm) served as the counterface material. Sliding tests were conducted under a normal load of 1 kgf (9.8 N) with a reciprocating amplitude of 5 mm and a sliding speed of 200 mm/min for a total duration of 60 min.

The wear rate (k) was calculated according to the Archard wear equation:

k=V/(F×L)

where V is the wear volume loss (mm^3^), F is the applied normal load (N), and L is the total sliding distance (m). Wear volume was determined by profilometry across the wear track.

### 2.4. Electrochemical Corrosion Testing

Corrosion resistance of the coatings was evaluated by potentiodynamic polarization measurements following ASTM G59-23 [27] in a 3.5 wt.% NaCl solution at room temperature. A conventional three-electrode cell was employed, consisting of the coated sample as the working electrode (exposed area 1 cm^2^), a saturated calomel electrode (SCE) as the reference electrode, and a platinum sheet as the counter electrode. Electrochemical measurements were performed using a PGSTAT302N potentiostat (Metrohm Autolab B.V., Utrecht, The Netherlands).

Prior to polarization, samples were immersed in the electrolyte for 1–2 h to achieve a stable open circuit potential (EOCP). Potentiodynamic polarization scans were conducted from −0.4 V to +0.4 V relative to EOCP at a scan rate of 1 mV/s. Corrosion potential (*E_corr_*) and corrosion current density (*I_corr_*) were determined by Tafel extrapolation of the polarization curves.

## 3. Results and Discussion

### 3.1. Microstructure

The microstructure of TiMoSiN coatings, including surface morphology, chemical composition, phase structure, and cross-sectional architecture, exhibits a strong dependence on nitrogen partial pressure. These microstructural characteristics are systematically presented below, with an emphasis on their interrelations.

#### 3.1.1. Surface Morphology

**Figure 2:**
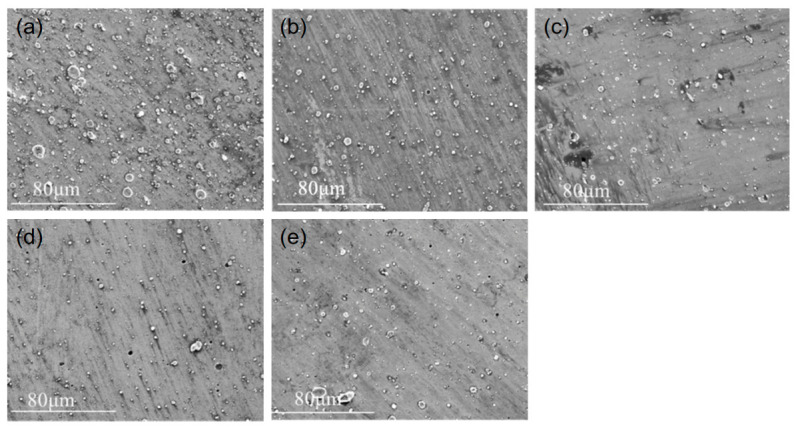
Surface morphology of TiMoSiN coatings deposited at various nitrogen partial pressures: (**a**) 0.2 Pa; (**b**) 0.6 Pa; (**c**) 1.0 Pa; (**d**) 1.3 Pa; (**e**) 1.7 Pa.

Figure 2 shows the surface morphology of TiMoSiN coatings deposited at various nitrogen partial pressures. All coatings exhibit the characteristic macroparticles inherent to the arc ion plating process. At a low nitrogen partial pressure of 0.2 Pa (Figure 2a), the coating surface displays numerous macroparticles with a wide size distribution. These macroparticles originate from localized melting of the target material due to excessive arc spot temperatures exceeding the melting point, creating micro-pools from which droplets are ejected and transported to the substrate [28,29].

As the nitrogen partial pressure increases from 0.2 to 1.0 Pa (Figure 2b,c), a progressive reduction in macroparticle density is observed. This phenomenon can be attributed to the formation of nitrides on the target surface at higher nitrogen partial pressures, which elevates the effective melting point and suppresses micro-pool formation [30]. Consequently, fewer droplets are emitted from the target, resulting in smoother coating surfaces. However, when the nitrogen partial pressure exceeds 1.3 Pa (Figure 2d,e), the coating surface exhibits occasional pit defects, likely caused by the detachment of poorly adhered macroparticles under intensified ion bombardment at higher gas pressures. These morphological variations affect mechanical and corrosion properties.

Quantitative roughness measurements (e.g., by profilometry) were not performed in this study. The discussion of surface morphology is therefore based on qualitative comparison of the SEM images, which consistently show a reduction in macroparticle density as the nitrogen pressure increases.

#### 3.1.2. Chemical Composition

The elemental compositions of TiMoSiN coatings analyzed by EDS are summarized in Table 2. With increasing nitrogen partial pressure from 0.2 to 1.7 Pa, the metallic elements Ti and Mo gradually decrease from 49.68 to 44.83 at.% and from 5.18 to 4.06 at.%, respectively, while the non-metallic elements Si and N increase from 0.24 to 2.64 at.% and from 44.90 to 48.47 at.%, respectively. The Mo/(Ti+Mo) atomic ratio also decreases from 0.0944 to 0.0830 with increasing pressure.

The decrease in Mo content with increasing nitrogen partial pressure can be explained by scattering phenomena in the plasma. Given the higher atomic mass of Mo compared to Ti, Mo species are more susceptible to scattering collisions with gas molecules, leading to a shorter mean free path and greater transport losses [31]. Higher nitrogen partial pressures further reduce the mean free path of all species, exacerbating scattering losses and preferentially reducing the deposition rate of heavier elements such as Mo.

Furthermore, the deviation between coating and target compositions, termed compositional segregation, arises from multiple factors inherent to the PVD process [9,31]: (1) melting point differences: Mo has a substantially higher melting point (2623 °C) than Ti (1668 °C), favoring Ti evaporation and enriching Ti in the vapor phase; (2) Mo possesses a lower saturated vapor pressure than Ti, resulting in higher ionization efficiency and preferential deposition under negative substrate bias; (3) resputtering effects: deposited material may be re-sputtered by incoming ions, with differential rates for different elements. These factors collectively result in Ti/Mo atomic ratios in the coatings (ranging from 6.1 to 8.8) substantially higher than the target ratio of 8.0.

Notably, the Si content increases significantly with nitrogen partial pressure, which plays a crucial role in phase evolution and grain refinement, as discussed in the following sections.

#### 3.1.3. Phase Structure

**Figure 3:**
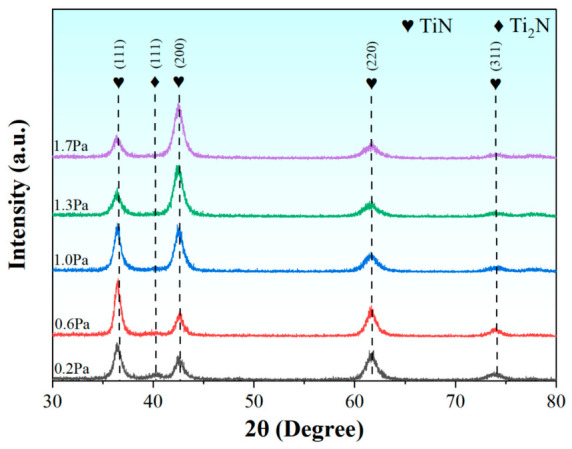
XRD patterns of TiMoSiN coatings deposited at various nitrogen partial pressures.

Grazing incidence X-ray diffraction (GIXRD) patterns of TiMoSiN coatings deposited at different nitrogen partial pressures are presented in Figure 3. All coatings predominantly exhibit face-centered cubic (fcc) structure characteristic of TiN (JCPDS No. 38-1420). At low nitrogen partial pressures (0.2 and 0.6 Pa), additional peaks corresponding to tetragonal Ti_2_N phase (JCPDS No. 17-0386) are detected, indicating incomplete nitridation under nitrogen-deficient conditions.

Notably, despite significant Mo and Si contents (Table 2), no diffraction peaks corresponding to Mo-containing phases (e.g., Mo, Mo_2_N, MoN) or Si-containing phases (e.g., Si_3_N_4_) are observed. This suggests that Mo atoms substitute for Ti in the TiN lattice, forming (Ti,Mo)N solid solutions, while Si likely segregates to grain boundaries as amorphous Si_3_N_4_, a common feature in Ti-Si-N nanocomposite systems [21,32]. Compared to standard TiN diffraction peaks, all reflections shift slightly toward lower diffraction angles, indicating lattice expansion due to incorporation of larger Mo atoms [8].

Nitrogen partial pressure significantly influences preferred orientation. At 0.2 and 0.6 Pa, coatings exhibit strong (111) preferred orientation. At 1.0 Pa, both (111) and (200) reflections appear with comparable intensity. Further increasing pressure to 1.3 and 1.7 Pa eliminates the (111) orientation, with (200) becoming the dominant texture. This evolution reflects the competition between surface energy and strain energy minimization during film growth [33]. At low pressures, energetic ion bombardment favors close-packed (111) planes; at higher pressures, reduced ion energy and increased nitrogen availability promote (200) orientation characteristic of stoichiometric TiN.

Concurrently, the full width at half maximum (FWHM) of the (111) and (200) reflections broadens with increasing nitrogen partial pressure, indicating a decrease in crystallite size. Using the Scherrer formula applied to the (111) and (200) diffraction peaks, the average crystallite size was estimated. The results show a decrease from approximately 14 nm at 0.2 Pa to about 8 nm at 1.7 Pa, confirming the grain refinement trend indicated by the broadening of XRD peaks.

**Figure 4:**
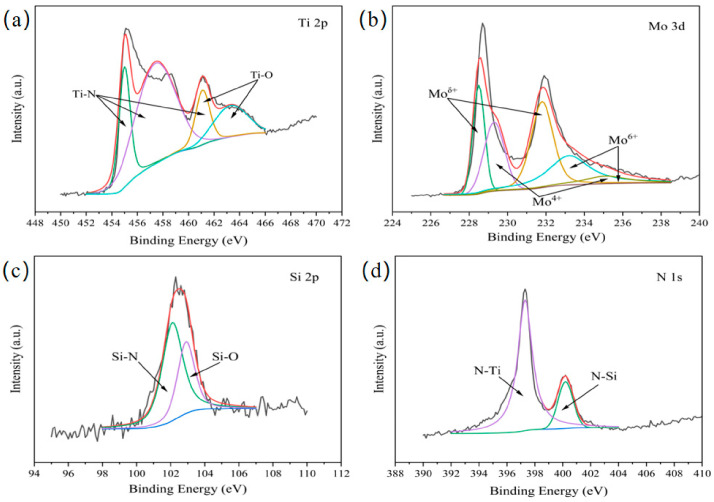
XPS spectra of the TiMoSiN coating deposited at 0.6 Pa: (**a**) Ti 2p, (**b**) Mo 3d, (**c**) Si 2p, and (**d**) N 1s.

To further elucidate the chemical bonding states of Si, X-ray photoelectron spectroscopy (XPS) was performed on the coating deposited at 0.6 Pa. As shown in Figure 4, the Si2p spectrum exhibits a peak at 102.1 eV, corresponding to Si–N bonds in Si_3_N_4_, and the N1s spectrum shows a peak at 400.2 eV, also attributed to N–Si bonds. These results directly confirm the formation of amorphous Si_3_N_4_ at grain boundaries, which is characteristic of the nc-TiN/a-Si_3_N_4_ nanocomposite structure. No other Si-containing crystalline phases are detected, consistent with the XRD results.

#### 3.1.4. Cross-Sectional Morphology

**Figure 5:**
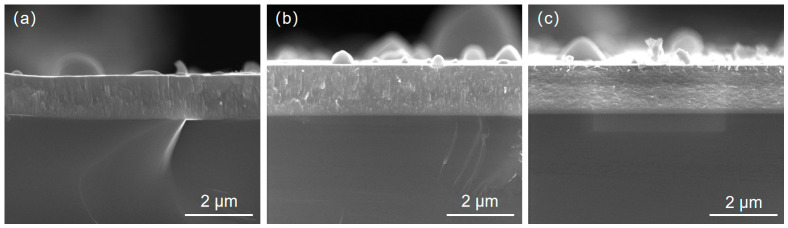
Cross-sectional microstructure of TiMoSiN coatings deposited at various nitrogen partial pressures: (**a**) 0.2 Pa; (**b**) 0.6 Pa; (**c**) 1.0 Pa.

Cross-sectional SEM images of TiMoSiN coatings deposited at various nitrogen partial pressures are shown in Figure 5. Consistent with the XRD observations, the coating microstructure evolves significantly with nitrogen partial pressure.

At 0.2 Pa (Figure 5a), the coating exhibits a distinct columnar structure characteristic of Zone T of the Thornton structure zone model [34,35], with columns extending through the entire coating thickness. At 0.6 Pa (Figure 5b), columnar growth becomes less defined, with intermittent fine-grained regions interrupting column continuity. At pressures higher than 1.0 Pa (Figure 5c), columnar growth completely disappears, yielding a dense, featureless fine-grained microstructure.

This microstructural evolution correlates strongly with increasing Si content (Table 2) and the formation of amorphous Si_3_N_4_ at grain boundaries. The amorphous Si_3_N_4_ phase acts as a grain growth inhibitor, disrupting epitaxial growth of TiN columns and promoting repeated nucleation of fine grains [21,32,36]. The transition from columnar to fine-grained microstructure is expected to significantly influence mechanical and tribological properties, as discussed in subsequent sections.

#### 3.1.5. Thickness and Deposition Rate

The thickness of the TiMoSiN coatings measured by the ball-cratering method is summarized in Table 3. With increasing nitrogen pressure, the thickness increases slightly from 2.55 μm at 0.2 Pa to 2.78 μm at 1.3 Pa, then decreases slightly to 2.77 μm at 1.7 Pa. The corresponding deposition rates (thickness divided by deposition time of 60 min) range from 0.042 μm/min to 0.047 μm/min. The slight increase in deposition rate at higher nitrogen pressures is attributed to enhanced ionization and greater nitrogen incorporation, which promote more efficient deposition of nitrogen containing species.

### 3.2. Mechanical Properties

**Figure 6:**
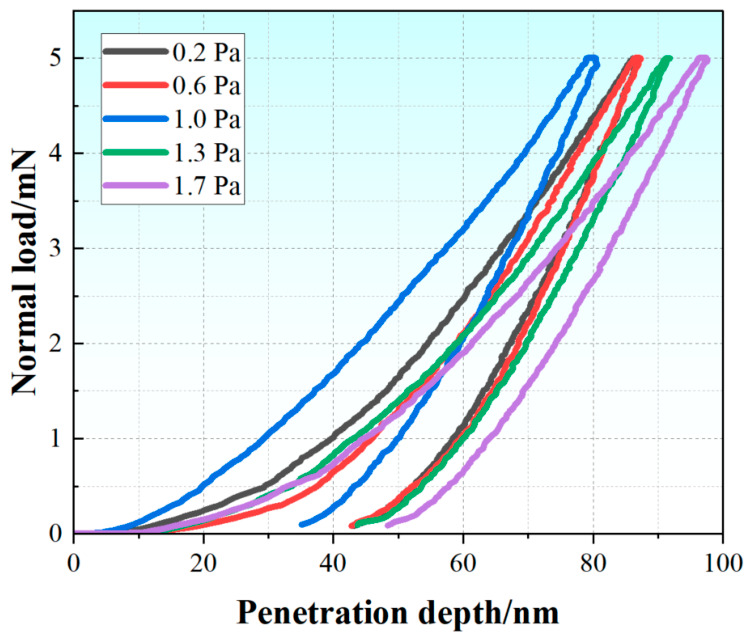
Representative nanoindentation load–displacement curves of TiMoSiN coatings deposited at various nitrogen partial pressures.

The mechanical properties of TiMoSiN coatings were evaluated by nanoindentation. Figure 6 shows representative load–displacement curves for coatings deposited at different nitrogen partial pressures. Under a maximum load of 5 mN, all indentation depths remain below 100 nm, which is less than 10% of the coating thickness. This ensures that substrate interference is negligible and that the measured hardness values represent the intrinsic properties of the coatings.

**Figure 7:**
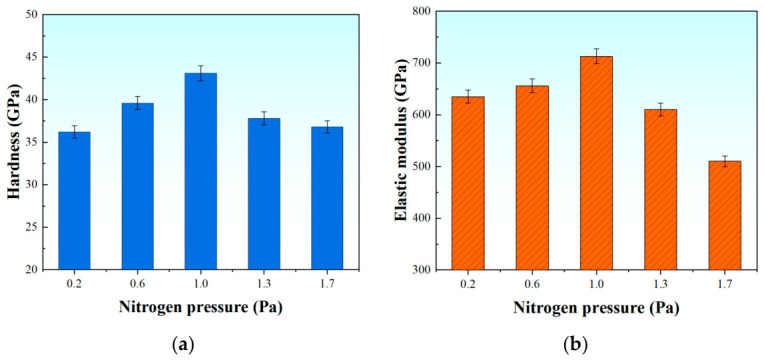
(**a**) Hardness and (**b**) elastic modulus of TiMoSiN coatings deposited at various nitrogen partial pressures.

Figure 7 presents the hardness and elastic modulus of TiMoSiN coatings as functions of nitrogen partial pressure. All coatings exhibit hardness exceeding 35 GPa, substantially higher than conventional TiN (about 15–25 GPa) and TiMoN coatings (about 30–35 GPa) prepared under comparable conditions [8,9,37,38]. Hardness follows a parabolic trend with increasing nitrogen partial pressure: it rises from 36.2 GPa at 0.2 Pa to a maximum of 43.1 GPa at 1.0 Pa, and then decreases to 38.4 GPa at 1.7 Pa. The maximum hardness of 43.1 GPa meets the criterion for superhard coatings (>40 GPa).

The enhanced hardness of TiMoSiN coatings relative to binary and ternary counterparts can be attributed to multiple synergistic strengthening mechanisms: (1) Solid-solution strengthening: Mo atoms substituting for Ti in the TiN lattice introduce local lattice distortions that impede dislocation motion, thereby increasing the stress required for plastic deformation [38]. (2) Second-phase strengthening: At low nitrogen partial pressures (0.2–0.6 Pa), the coexistence of TiN and Ti_2_N phases provides additional strengthening through the Koehler effect—the resistance to dislocation motion across phase boundaries with different shear moduli. Moreover, Ti_2_N (tetragonal structure) has a higher intrinsic hardness than fcc-TiN, contributing to composite hardening [8,37]. (3) Nanocomposite strengthening: The formation of amorphous Si_3_N_4_ at grain boundaries creates a nanocomposite structure consisting of nanocrystalline (Ti,Mo)N embedded in an amorphous matrix. Such structures are known to exhibit superhardness because grain boundary sliding and dislocation activity are restricted [39]. Residual stress analysis was not carried out in this study; therefore, the potential contribution of residual stresses to the measured hardness and tribological behavior is not discussed. 

The hardness maximum at 0.6–1.0 Pa represents an optimal balance of these strengthening mechanisms. Below 0.6 Pa, limited Si incorporation restricts nanocomposite formation; above 1.0 Pa, the disappearance of Ti_2_N and excessive amorphous Si_3_N_4_ lead to hardness degradation. The elastic modulus (E) follows a trend similar to that of hardness, ranging from 500 to 750 GPa, with the maximum also observed at 1.0 Pa.

### 3.3. Tribological Performance

**Figure 8:**
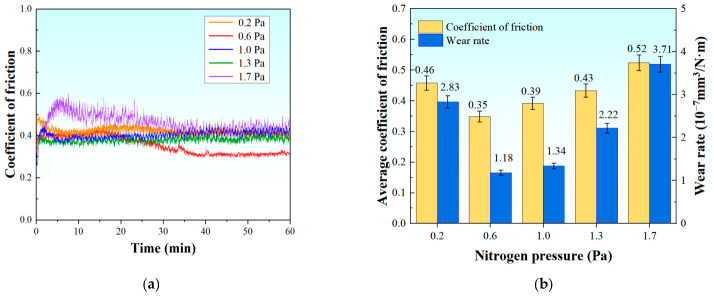
(**a**) Friction coefficient curves and (**b**) average friction coefficient and wear rate of TiMoSiN coatings deposited at various nitrogen partial pressures.

The tribological performance of TiMoSiN coatings was evaluated by reciprocating sliding tests against Al_2_O_3_ counterbodies under ambient conditions. Figure 8a presents the friction coefficient curves as a function of sliding time, and Figure 8b summarizes the average friction coefficients and wear rates.

At 0.2 Pa, the coating exhibits the poorest tribological properties among all coatings deposited below 1.0 Pa, with an average friction coefficient of 0.458 and a wear rate of 2.83 × 10^−7^ mm^3^/(N·m). The friction curve shows an extended running-in stage of approximately 30 min, after which friction stabilizes at about 0.4. This poor performance is primarily attributed to the rough surface morphology at low pressure, which contains abundant macroparticles. During initial sliding, these macroparticles fragment and generate wear debris, exacerbating three-body abrasion and prolonging the running-in process [40]. Additionally, the columnar microstructure at 0.2 Pa (Figure 5a) provides less resistance to crack propagation and debris detachment [41]. Consequently, low nitrogen partial pressure is detrimental to tribological performance.

Both coatings deposited at 0.6 Pa and 1.0 Pa exhibit significantly better tribological properties than the 0.2 Pa coating, with the best overall performance achieved at 0.6 Pa.

**Figure 9:**
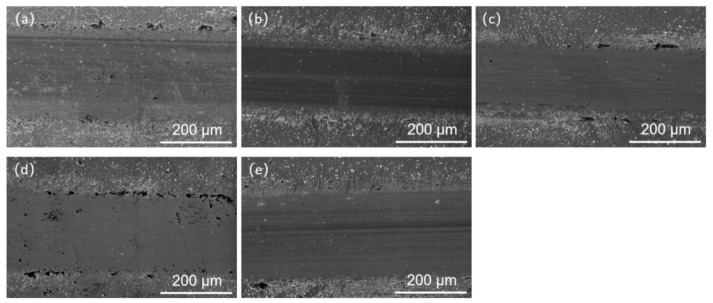
Wear tracks of TiMoSiN coatings deposited at various nitrogen partial pressures: (**a**) 0.2 Pa; (**b**) 0.6 Pa; (**c**) 1.0 Pa; (**d**) 1.3 Pa; (**e**) 1.7 Pa.

At 0.6 Pa, the coating shows the lowest average friction coefficient (0.349) and the lowest wear rate (1.08 × 10^−7^ mm^3^/(N·m)) among all coatings. The friction curve exhibits a remarkably brief running-in stage (2–3 min), followed by steady-state friction at about 0.4. Notably, after approximately 25 min, friction progressively decreases to about 0.3 and remains stable until the end of the test. This friction reduction suggests the gradual formation of lubricious tribo-layers, potentially MoO_3_ on the contact surface. This observation is consistent with the well-established role of Mo in reducing friction through the formation of lubricious MoO_3_ during sliding [8]. The wear track is the narrowest (about 100 μm) and exhibits mild abrasive wear characteristics (Figure 9b). In contrast, the wear tracks at 0.2 Pa (Figure 9a) are wide grooved, while those at 1.3 Pa and 1.7 Pa (Figure 9d,e) also show wide wear tracks.

At 1.0 Pa, the coating also demonstrates favorable tribological properties, with an average friction coefficient of 0.392 and a wear rate of 1.59 × 10^−7^ mm^3^/(N·m). Although this coating achieves the highest hardness (43.1 GPa), its friction coefficient and wear rate are slightly higher than those of the 0.6 Pa coating. This may be due to the preferred orientation transition from (111) to (200) (Figure 3) and the more pronounced abrasive cutting action of the harder surface against the Al_2_O_3_ counterface, which could delay the formation of stable tribo-layers [42].

Overall, the 0.6–1.0 Pa range provides a favorable combination of reduced surface defects, optimized phase composition, and appropriate hardness, leading to superior tribological performance.

When the nitrogen partial pressure increases further to 1.3 Pa and 1.7 Pa, the tribological properties deteriorate significantly. At 1.3 Pa, the average friction coefficient rises to 0.433 and the wear rate to 2.22 × 10^−7^ mm^3^/(N·m). At 1.7 Pa, the performance becomes the poorest among all coatings, with an average friction coefficient of 0.524 and a wear rate of 3.71 × 10^−7^ mm^3^/(N·m). The friction curve at 1.7 Pa exhibits substantial initial fluctuations, with friction rising from 0.3 to 0.55 within the first 5 min, followed by a gradual decrease to about 0.42 after 30 min, indicating a prolonged and severe running-in stage.

The degradation at high nitrogen partial pressures is attributed to several factors. First, the hardness decreases from its maximum at 1.0 Pa to 41.2 GPa at 1.3 Pa and 38.4 GPa at 1.7 Pa (Figure 7), reducing the coating’s resistance to plastic deformation and abrasive wear. Second, the preferred orientation becomes exclusively (200) at high pressures, which is associated with lower hardness and potentially different tribo-chemical reactivity [43]. Wear track analysis (Figure 9d,e) reveals a transition in wear mechanism from abrasive to adhesive wear. A detailed comparison across different nitrogen partial pressures illustrates this evolution clearly. At 0.2 Pa, the wear track exhibits deep continuous grooves and abundant loose debris, characteristic of severe abrasive wear. At 0.6 Pa, the track is narrow and smooth, with only mild abrasive scratches. As the pressure increases to 1.0 Pa and above, the grooves become less pronounced, but patches of adhered material and smearing appear, marking a gradual transition from abrasive to adhesive wear. This transition is most evident at 1.7 Pa, where the track exhibits extensive material transfer and smearing.

The tribological performance of TiMoSiN coatings exhibits a strong dependence on nitrogen partial pressure, following a “U-shaped” trend. Too low a pressure (0.2 Pa) leads to rough surfaces with abundant macroparticles and a columnar microstructure, causing high friction and wear. Too high a pressure (≥1.3 Pa) results in decreased hardness, excessive amorphous phase formation, and a transition to adhesive wear, also degrading performance. An optimal window exists at intermediate pressures (0.6–1.0 Pa), where the coating achieves a balanced combination of smooth surface morphology, high hardness, favorable phase composition, and the ability to form lubricious tribo-layers. Among these, the coating deposited at 0.6 Pa exhibits the best overall tribological performance, with the lowest friction coefficient and wear rate.

**Figure 10:**
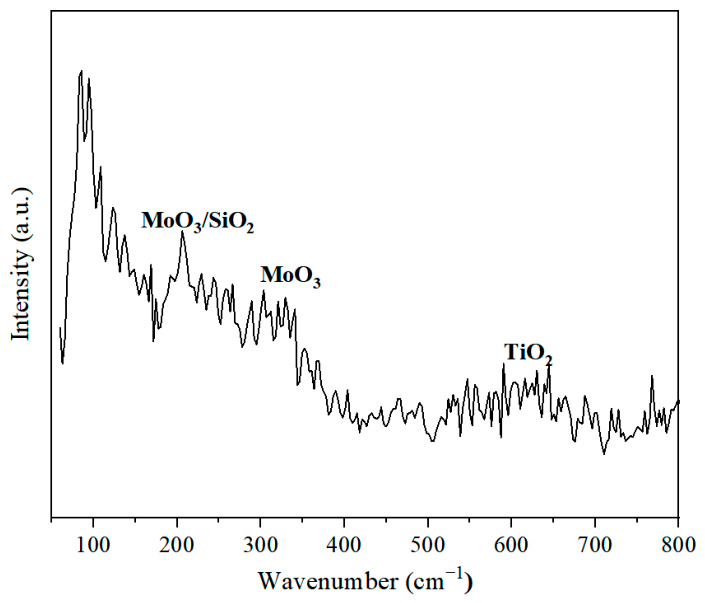
Raman spectrum of the wear track on the TiMoSiN coating deposited at 0.6 Pa, showing peaks of MoO_3_ (216 cm^−1^, 319 cm^−1^) and SiO_2_ (609 cm^−1^).

To verify the proposed formation of lubricious tribo-layers, Raman spectroscopy was performed on the wear track of the coating deposited at 0.6 Pa. As shown in Figure 10, the Raman spectrum reveals distinct peaks at 216 cm^−1^ and 319 cm^−1^, characteristic of MoO_3_, and a peak at 609 cm^−1^ corresponding to SiO_2_. These results directly confirm the presence of MoO_3_ and SiO_2_ on the worn surface, which account for the observed friction reduction during steady-state sliding.

### 3.4. Corrosion Resistance

**Figure 11:**
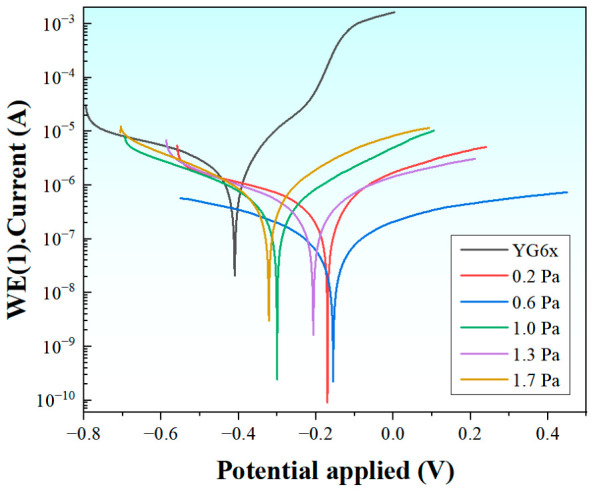
Polarization curves of uncoated YG6x and TiMoSiN coatings deposited at various nitrogen partial pressures.

The corrosion behavior of TiMoSiN coatings and the uncoated YG6x substrate was evaluated by potentiodynamic polarization in 3.5 wt.% NaCl solution. Figure 11 presents the polarization curves, and Table 4 summarizes the electrochemical parameters derived from Tafel extrapolation.

The uncoated YG6x substrate exhibits the poorest corrosion resistance, with the most negative corrosion potential (*E_corr_* = −406 mV vs. SCE) and highest corrosion current density (*i_corr_* = 2.59 × 10^−6^ A·cm^−2^). All TiMoSiN coatings demonstrate substantially improved corrosion resistance, with more noble E_corr_ values (ranging from −323 to −152 mV) and reduced i_corr_ (from 5.97 × 10^−7^ to 8.99 × 10^−8^ A·cm^−2^). This improvement confirms that TiMoSiN coatings effectively act as physical barriers against corrosive media penetration.

The corrosion resistance varies non-monotonically with nitrogen partial pressure: it first improves from 0.2 Pa to 0.6 Pa, then progressively deteriorates at 1.0 Pa and higher pressures. This trend is correlated with the coatings’ surface defect density, Mo content, and microstructure observed in this study.

#### 3.4.1. Inferior Corrosion Resistance at Low Pressure

At 0.2 Pa, the coating exhibits a corrosion current density of 3.92 × 10^−7^ A·cm^−2^, which is higher than that at 0.6 Pa. The primary reason is the high density of macroparticles on the surface (Figure 2a). These macroparticles are inherent to arc ion plating at low nitrogen partial pressure. They create micro-defects such as pinholes and weakly adhered interfaces, which serve as preferential pathways for electrolyte penetration [44]. Once the electrolyte reaches the substrate, galvanic corrosion between the coating and the cemented carbide substrate can occur, accelerating material loss. Although the Mo content at 0.2 Pa is the highest among all coatings (5.18 at.%, Table 2), the beneficial effect of Mo on corrosion resistance, widely recognized for its ability to stabilize passive films in chloride environments, is overwhelmed by the high defect density [45]. This is consistent with the well-known principle that coating barrier performance is primarily determined by the most defective sites rather than by average composition.

#### 3.4.2. Optimal Corrosion Resistance at 0.6 Pa

The coating deposited at 0.6 Pa achieves the best corrosion resistance, with the highest *E_corr_* (−152 mV) and the lowest *i_corr_* (8.99 × 10^−8^ A·cm^−2^), approximately 4.4 times lower than that at 0.2 Pa and 29 times lower than the uncoated substrate.

The mutually reinforcing factors observed in this study that contribute to this optimum are reduced defects and sufficient Mo content. At 0.6 Pa, the surface macroparticle density is substantially lower than at 0.2 Pa (Figure 2b). This reduces the number of corrosion initiation sites. Moreover, the cross-sectional microstructure at 0.6 Pa (Figure 5b) shows a transition from the fully columnar structure seen at 0.2 Pa (Figure 5a) to a less columnar, intermittently fine-grained morphology. Columnar boundaries are known to be fast diffusion paths for corrosive species [46]; their disruption at 0.6 Pa enhances barrier integrity. The Mo content at 0.6 Pa is 4.84 at.%. While slightly lower than at 0.2 Pa, it remains at a level that can effectively promote corrosion resistance. The present study does not directly analyze the passive film composition, but the clear improvement in *E_corr_* and *i_corr_* at 0.6 Pa compared to 0.2 Pa indicates that the combination of moderate Mo content and lower defect density is more beneficial than high Mo content with high defect density.

Thus, 0.6 Pa represents an optimal balance where defect density is sufficiently low, Mo content is still adequate, and the microstructure limits rapid diffusion.

#### 3.4.3. Deterioration at Higher Pressures

As nitrogen partial pressure increases from 1.0 to 1.7 Pa, the corrosion resistance progressively worsens. At 1.0 Pa, *i_corr_* increases to 3.48 × 10^−7^ A·cm^−2^ (about 4 times higher than at 0.6 Pa), and at 1.7 Pa, *i_corr_* reaches 5.97 × 10^−7^ A·cm^−2^.

The deterioration can be explained by two trends observed in this study: (1) Decreasing Mo content: With increasing pressure, the Mo content monotonically decreases from 4.84 at.% at 0.6 Pa to 4.06 at.% at 1.7 Pa (Table 2). Lower Mo content reduces the coating’s ability to form a protective passive film, making it more susceptible to chloride attack. (2) Microstructural changes: At pressures higher than 1.0 Pa, the cross-sectional microstructure becomes fully fine-grained and featureless (Figure 5c). While this eliminates columnar boundaries, the high density of grain boundaries can provide alternative diffusion pathways for corrosive species. Additionally, at 1.3 Pa and 1.7 Pa, occasional pit defects appear on the surface (Figure 2d,e), which can act as localized corrosion initiation sites. (3) Effect of excessive Si: Silicon predominantly forms amorphous Si_3_N_4_ at grain boundaries. At moderate content (e.g., 0.6 Pa), this amorphous phase can seal diffusion pathways and improve corrosion resistance. However, at higher nitrogen pressures (≥1.3 Pa), the increased Si content may lead to an excessive volume fraction of amorphous Si_3_N_4_ and a high density of amorphous/crystalline interfaces, which can act as preferential pathways for corrosive species, further accelerating corrosion.

It is worth noting that while the surface macroparticle density continues to decrease at higher pressures, the negative effects of reduced Mo content and microstructural changes outweigh this benefit. Consequently, the corrosion resistance does not improve further but instead declines.

## 4. Conclusions

From the systematic investigation of nitrogen partial pressure (0.2–1.7 Pa) on arc-ion-plated TiMoSiN coatings, the following conclusions are drawn:

(1) Nitrogen partial pressure is an effective control parameter for tuning the phase composition, preferred orientation, and microstructure. High pressure (≥1.0 Pa) promotes a single fcc-TiN phase with (200) orientation and eliminates columnar growth, while low pressure (≤0.6 Pa) yields mixed TiN+Ti_2_N phases with (111) orientation and a columnar structure.
(2) All coatings exhibit high hardness (36.2–43.1 GPa), with the maximum (43.1 GPa) at 1.0 Pa. However, the best tribological performance (lowest friction coefficient of 0.349 and wear rate of 1.08 × 10^−7^ mm^3^/(N·m)) is achieved at 0.6 Pa, indicating that hardness alone does not dictate wear resistance; surface defect density and the ability to form lubricious tribo-layers are equally important.
(3) The optimal corrosion resistance (*E_corr_* = –152 mV, *i_corr_* = 8.99 × 10^−8^ A·cm^−2^) is also obtained at 0.6 Pa, owing to a balanced combination of reduced macroparticles, sufficient Mo content (4.84 at.%), and a partially disrupted columnar microstructure.
(4) Excessively low (0.2 Pa) or high (≥1.3 Pa) nitrogen partial pressure degrades both tribological and corrosion performance. Therefore, a moderate nitrogen partial pressure of 0.6–1.0 Pa is recommended for arc-ion-plated TiMoSiN coatings demanding high wear and corrosion resistance, with the 0.6 Pa condition providing the overall best balance. This makes the coating a promising candidate for industrial applications requiring integrated wear resistance, low friction, and corrosion protection under aggressive conditions.

## Figures and Tables

**Table 1:** Process parameters for TiMoSiN coating deposition by arc ion plating.

Process	Ion Bombarding	Cr Interlayer	TiMoSiN Layer
Time (min)	10	10	60
Cr arc current (A)	75	75	-
TiMoSi arc current (A)	-	-	100
Negative bias (V)	500	100	150
Duty cycle (%)	60	60	60
Ar flow	Open	Open	-
N_2_ flow	-	-	Open
Pressure (Pa)	0.2	0.4	0.2/0.6/1.0/1.4/1.7
Temperature (°C)	250	250	250

**Table 2:** Elemental compositions of TiMoSiN coatings deposited at various nitrogen partial pressures.

Coatings	Nitrogen Partial Pressure (Pa)	Elemental Composition (at%)				
		Ti	Mo	Si	N	Mo/(Ti+Mo)
TiMoSiN	0.2	49.68	5.18	0.24	44.90	0.0944
	0.6	48.46	4.84	1.03	45.00	0.0908
	1.0	45.48	4.27	2.05	48.20	0.0858
	1.3	45.24	4.23	2.27	48.26	0.0855
	1.7	44.83	4.06	2.64	48.47	0.0830

**Table 3:** Thickness and deposition rate of TiMoSiN coatings deposited at different nitrogen pressures.

Nitrogen Partial Pressure (Pa)	Thickness (μm)	Deposition Rate (μm/min)
0.2	2.55	0.042
0.6	2.59	0.044
1.0	2.75	0.046
1.3	2.78	0.047
1.7	2.77	0.046

**Table 4:** Electrochemical parameters of TiMoSiN coatings and uncoated YG6x substrate.

Sample	*E_corr_* (mV vs. SCE)	*i_corr_* (A·cm^−2^)
YG6x (uncoated)	−406	2.59 × 10^−6^
0.2 Pa	−170	3.92 × 10^−7^
0.6 Pa	−152	8.99 × 10^−8^
1.0 Pa	−205	3.48 × 10^−7^
1.3 Pa	−299	4.67 × 10^−7^
1.7 Pa	−323	5.97 × 10^−7^

## Data Availability

The original contributions presented in this study are included in the article. Further inquiries can be directed to the corresponding authors.
